# Intra‐oesophageal invasion of thymoma

**DOI:** 10.1002/rcr2.485

**Published:** 2019-09-05

**Authors:** Makiko Ozawa, Toshirou Fukushima, Takuro Noguchi, Takashi Kobayashi, Nodoka Sekiguchi, Tomonobu Koizumi

**Affiliations:** ^1^ Second Department of Internal Medicine Shinshu University Graduate School of Medicine Matsumoto Japan; ^2^ Department of Comprehensive Cancer Therapy Shinshu University School of Medicine Matsumoto Japan

**Keywords:** Invasive thymoma, mediastinal tumour, oesophagus

## Abstract

This is the first report of a thymoma developing with unusual invasion into the oesophageal lumen.

## Clinical Image

**Figure 1:**
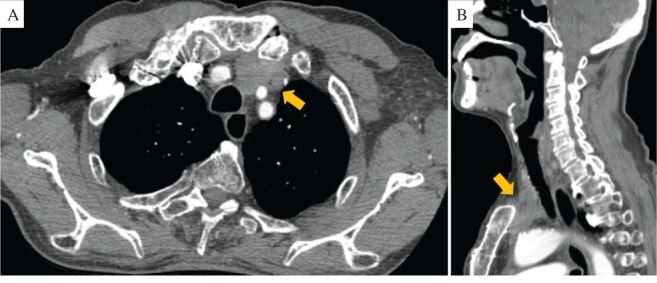
Chest computed tomography (CT) showed a tumour in anterior mediastinum and the mass had remained unchanged over the past decade as determined on serial chest CT examinations.

**Figure 2:**
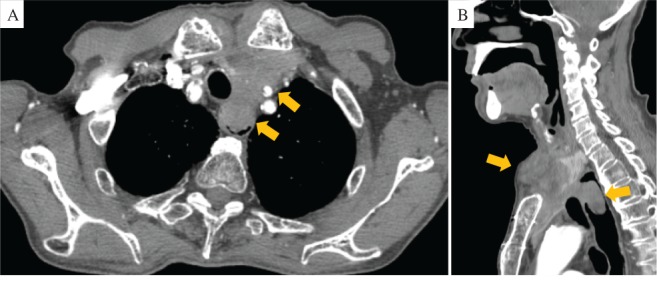
Recent chest computed tomography showed that the mass progressed rapidly and extended into the oesophageal lumen.

**Figure 3:**
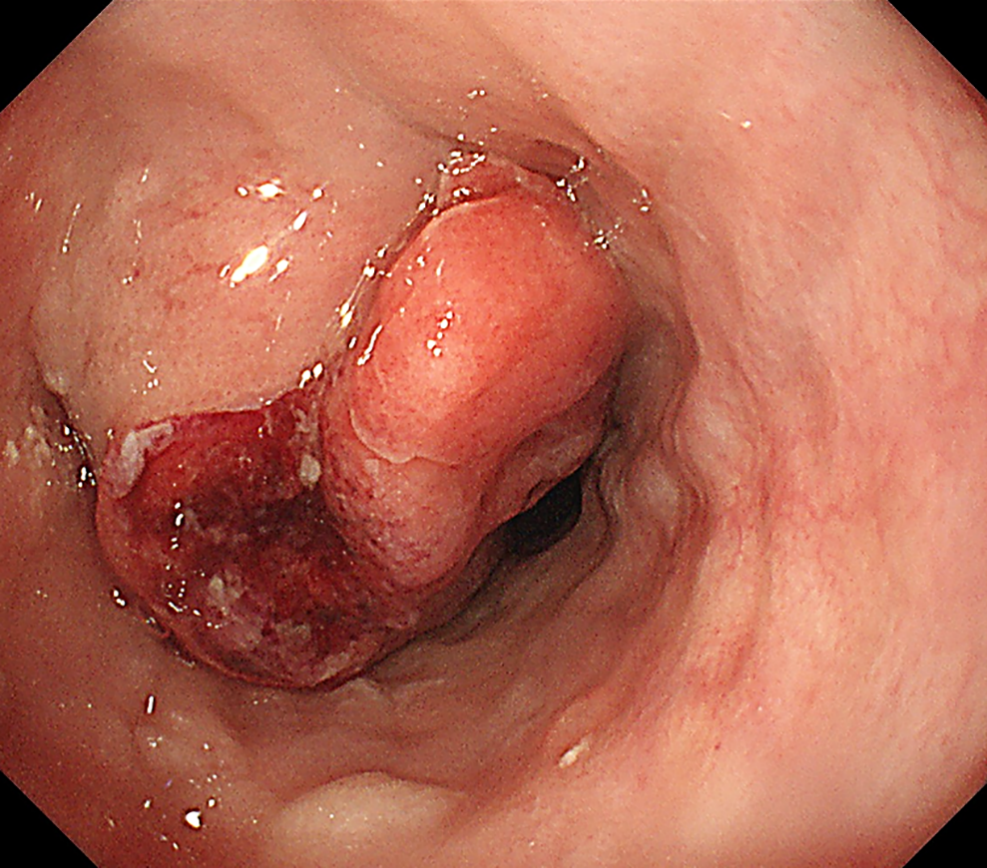
Endoscopic examination revealed a submucosal mass on the upper oesophagus.

A 79‐year‐old man diagnosed and treated for myasthenia gravis and thymoma since 1997 was admitted to our hospital in 2019 due to a rapidly progressing anterior mediastinal mass. At the time of initial diagnosis, thoracic surgery was performed but failed to remove the anterior mass because of ventricular fibrillation during the operation. He was treated with prednisolone (5–10 mg/day) and tacrolimus (2–3 mg/day) after radiotherapy (60 Gy) for the mass. The anterior mass had remained unchanged over the past decade as determined on serial chest computed tomography (CT) examinations (Fig. 1). However, the mass progressed rapidly and extended into the oesophageal lumen (Fig. 2). Endoscopic examination revealed a submucosal mass on the upper oesophagus (Fig. 3). Physical examination revealed an elevated subcutaneous mass (25 mm in size) in lower and mid‐anterior neck. He had complained of hoarseness since the initial diagnosis but no clinical symptoms such as dysphagia were observed. Percutaneous tumour biopsy was performed and histopathological examination revealed a World Health Organization type B3 thymoma. Three cycles of carboplatin plus paclitaxel were done but failed to reduce the mass. Several cases of intrabronchial spread were reported 1, 2. Ko et al. described a case of oesophageal submucosal tumour due to invasive thymoma 3. However, to our knowledge, this is the first report of a thymoma developing with such unusual invasion into the oesophageal lumen.

### Disclosure Statement

Appropriate written informed consent was obtained for publication of this case report and accompanying images.
